# Comparison of Intraoperative and Postoperative Outcomes With Skin Flaps Raised Using Either Harmonic Scalpels or Electrocautery During Modified Radical Mastectomy

**DOI:** 10.7759/cureus.62320

**Published:** 2024-06-13

**Authors:** Shubham Samal, Radhakrishna Ramchandani, Debajyoti Mohanty

**Affiliations:** 1 General Surgery, All India Institute of Medical Sciences, Raipur, Raipur, IND

**Keywords:** modified radical mastectomy, seroma, flap necrosis, electrocautery, harmonic scalpel

## Abstract

Background: Breast cancer is one of the most common malignancies in women. Hence, its treatment has become our utmost priority in developing countries like India. Modified radical mastectomy (MRM) has traditionally been used as the standard of care for early-stage invasive breast carcinoma and still is the most commonly used surgical treatment for carcinoma breast.

Aim: The study compared the incidence of intraoperative and postoperative outcomes with skin flaps raised using a harmonic scalpel versus those raised using electrocautery.

Methods: Sixty women with biopsy-proven breast cancer who had to undergo MRM were randomly assigned to undergo skin flap raising during mastectomy by using electrocautery or harmonic scalpel. Thirty patients had surgery with electrocautery (Group 1) and 30 with a harmonic scalpel (Group 2) by the same surgical team.

Results: The mean operative time was significantly longer with harmonic scalpel when compared to that with electrocautery (140.67 ± 28.55 vs. 122.00 ± 19.16 mins, P =0.004). The amount of intraoperative blood loss (178.33 ± 21.06 vs 138.50 ± 28.53 mL P = 0.001) was less in the group operated with the harmonic scalpel, which was statistically significant. There was no significant difference between the groups regarding total drainage content (310.83 ± 88.93 vs 298.20 ± 127.87 mL, P = 0.659), drain duration (6.83 ± 0.75 vs 7.43 ± 2.27 days, p=0.174), seroma (3.3% vs. 0%) wound infection (3.3% vs 0%), flap necrosis (16.7% vs. 3.3%, P = 0.195), duration of hospital stays (8.57 ± 0.77 vs 8.43 ± 1.61 days, p=0.684).

Conclusion: Harmonic scalpels have a few advantages over electrocautery, but are not cost-effective.

## Introduction

Breast cancer is the most common malignancy in women around the world, accounting for 25% of all cancers in women [1]. There are various modalities available for the treatment of breast cancer, varying from surgery to chemotherapy and radiotherapy. Surgical treatment includes mastectomy, breast-conserving surgery (BCT), and modified radical mastectomy (MRM). The objective of mastectomy is to remove the tumor-containing breast tissue along with the entire breast ductolobular tissue. Breast cancer usually develops after a series of epithelial changes in the terminal ductolobular unit (TDLU) [2]. Flap development in MRM, either with knife or electrocautery, is associated with increased blood loss, seroma, wound infection, flap necrosis, hematoma, and prolonged axillary drainage. Operative morbidity associated with MRM using a knife or electrocautery is between 30% and 50% [3].

The incidence of skin flap-related complications in patients with MRM is 5-30%. Ultrasonic dissection with a harmonic scalpel is a safe alternative tool for surgical dissection and hemostasis to electrocautery [3,4]. Compared to monopolar diathermy, harmonic dissection has several advantages, including lack of eschar formation over the blade, the narrow circumference of collateral thermal damage to surrounding tissue, no smoke, and no stimulation of motor nerves in the axilla. It cannot be used in patients with pacemakers [5,6]. Ultrasonic dissection using a harmonic scalpel uses technology that produces a balanced sinusoidal (or harmonic) ultrasonic wave. This is coupled to the metallic rod of the device, and the wave motion is converted into high-frequency mechanical motion at the tip of a blade located at the end of the rod. The blade can then cut and coagulate tissue precisely and controlled with minimal lateral thermal damage. The coagulation is provided by generating high temperatures at the probe tip, resulting in necrosis and even charring [7].

Few studies have compared the incidence of flap necrosis between the two methods. Although other studies have increased harmonic scalpels' use in MRM, it is not clearly understood. To compare the two groups, our study also considers various outcomes, like intraoperative blood loss, duration of operative time, amount of drain output, and duration of hospital stay.

## Materials and methods

The study was conducted in the Department of General Surgery at All India Institute of Medical Sciences (AIIMS), Raipur, Chhattisgarh. Clearance from the Institution Ethics Committee-Human Research (IEC-HR) was obtained before the recruitment of patients into the study protocol. After being approved by the IEC, AIIMS, Raipur, Chhattisgarh, with approval number AIIMSRPR/IEC/2019/363, the study was conducted for one year. All patients with biopsy-proven carcinoma of the breast who were planned for surgery were included and divided among the two study groups. In the available fixed time for this thesis work, we included a minimum of 60 patients (30 in each group).

Inclusion criteria included female patients aged more than 18 years with biopsy-proven carcinoma breast or phyllodes tumor. Exclusion criteria included any patient with a previous history of breast surgery, completion mastectomy, and axillary nodal dissection following prior lumpectomy, breast tuberculosis, any coexisting breast skin infections (dermatitis, eczema), any immuno-compromised patients, any history of breast injuries or burns, pregnant women, patients who were not fit for anesthesia and patients with advanced breast malignancies that were not operable.

Methodology

**Figure 1:**
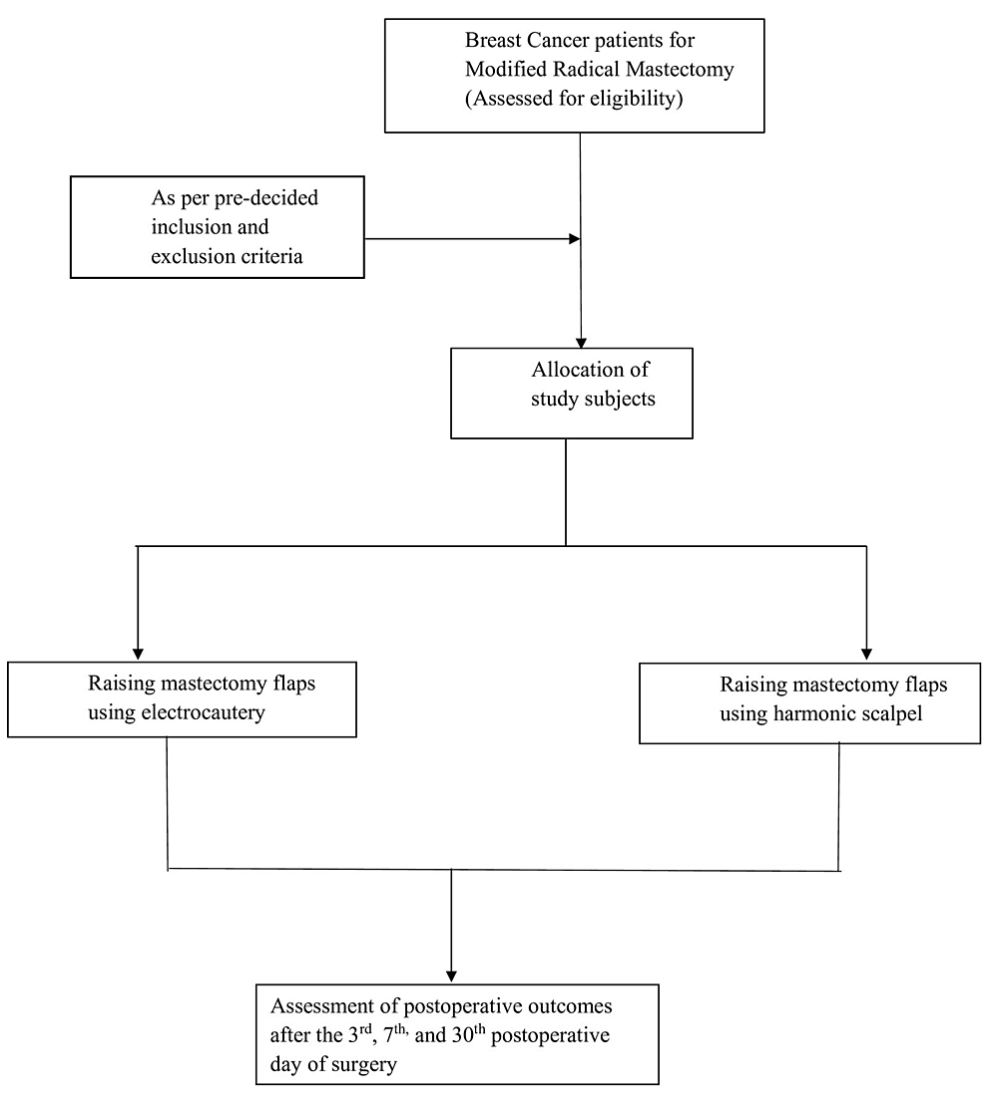
Flow diagram of participants. Shows the selection of patients and follow-up after surgery.

All the female patients who were diagnosed with carcinoma breast and satisfied the inclusion criteria were enrolled in the study. The patients who had completed neoadjuvant chemotherapy were also included. Detailed information regarding the patient's socio-demographic characteristics (age, gender, marital status, urban or rural area of residence, occupation) was obtained. Informed consent was taken from all the patients. Preanesthetic checkups and medical fitness were obtained. Patients were divided into two groups by alternate allocation; the first group (Group 1) included 30 females who underwent MRM using conventional electrocautery (monopolar diathermy), and the second group (Group 2) consisted of 30 females who underwent MRM using ultrasonic dissection (harmonic scalpel). Figure 1 shows the process of patient selection in different groups and their follow-up periods.

Operative procedure

The operative procedure was performed under general anesthesia. The starting time of the surgery was noted. After the patient had been positioned, cleaned, and draped, a skin incision was made with a scalpel. In Group 1, electrocautery was used to raise skin flaps. The time after raising skin flaps (upper and lower) was noted. In Group 2, the skin flaps were raised using a harmonic scalpel. The dissection for raising of upper and lower flaps was achieved by seal and cut mode of the harmonic scalpel. In both groups, the same technique for mastectomy along with axillary dissection was done using monopolar and bipolar diathermy. Two closed suction drains were placed. The skin was approximated using nonabsorbable sutures or staples. The antiseptic dressing was done regularly. The time between the first incision and the last suture or stapler (measured in minutes) was considered as the total operating time. Time taken for raising the skin flap was noted. Intraoperative blood loss was estimated by calculating the amount of blood in the suction container and several mops used for surgery. The total drain output was recorded regularly (in ml). The drains were removed when the amount of drain output was <30 ml/24 hrs. Each drain was removed on two different days based on the output. Adequate analgesics and antibiotics were given postoperatively.

**Figure 2:**
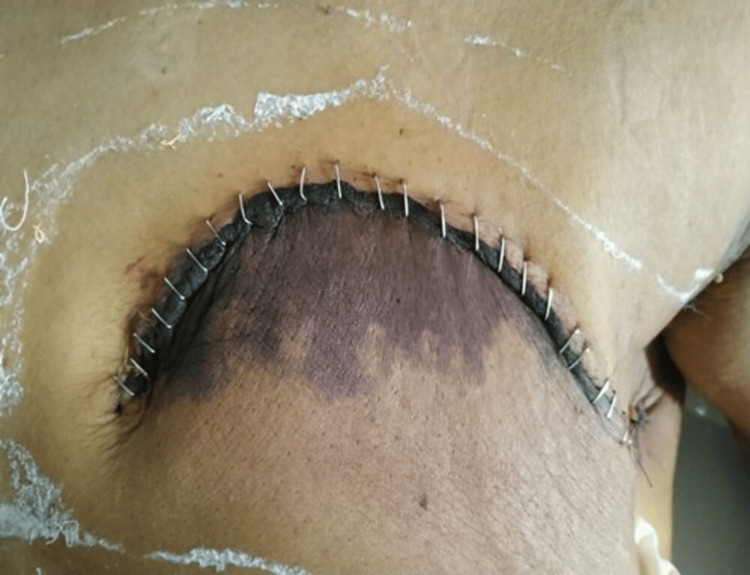
Showing lower flap necrosis. Taken from the study subject.

**Figure 3:**
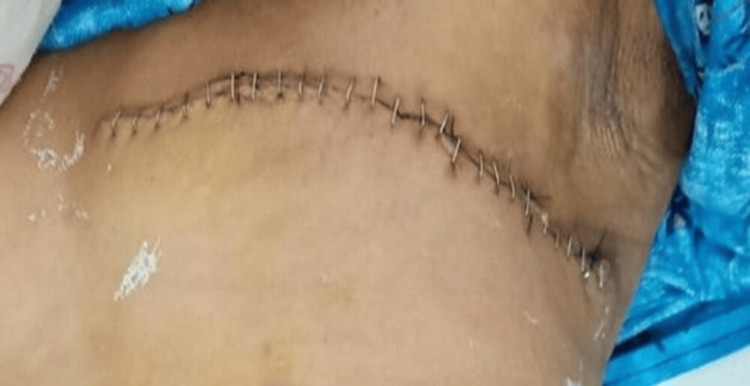
Showing no flap necrosis. Taken from the study subject.

The wound was inspected on days 3, 7, and 30 post-surgery. Postoperative complications including seroma, flap necrosis surgical site infection, and upper limb lymphedema of the operated side, were evaluated during the hospital stay and at follow-up. In the study, multiple aspirations along with pressure bandages were done for patients with seroma formation. The patients with flap necrosis and wound infections were treated with systemic and/or local antibiotics, regular dressings, and if required, debridement. Wounds were later closed by secondary suturing. Figure 2 and Figure 3 show the difference in identifying flap necrosis versus normal skin conditions in the postoperative period.

The patients with surgical site infections were treated with antibiotics according to culture and sensitivity reports and with aseptic daily dressing. The duration of hospital stay (days) of the patient after admission was noted. The follow-up of the patient was carried out till one month post-surgery. All the patients were advised for limb physiotherapy of the affected side in the postoperative period and also on discharge. The presence of surgical site infection is diagnosed by the presence of any purulent discharge from the surgical site or the patient has at least one of the following signs and symptoms: localized pain or tenderness, localized swelling, erythema, and local rise in temperature.

## Results

**Figure 4:**
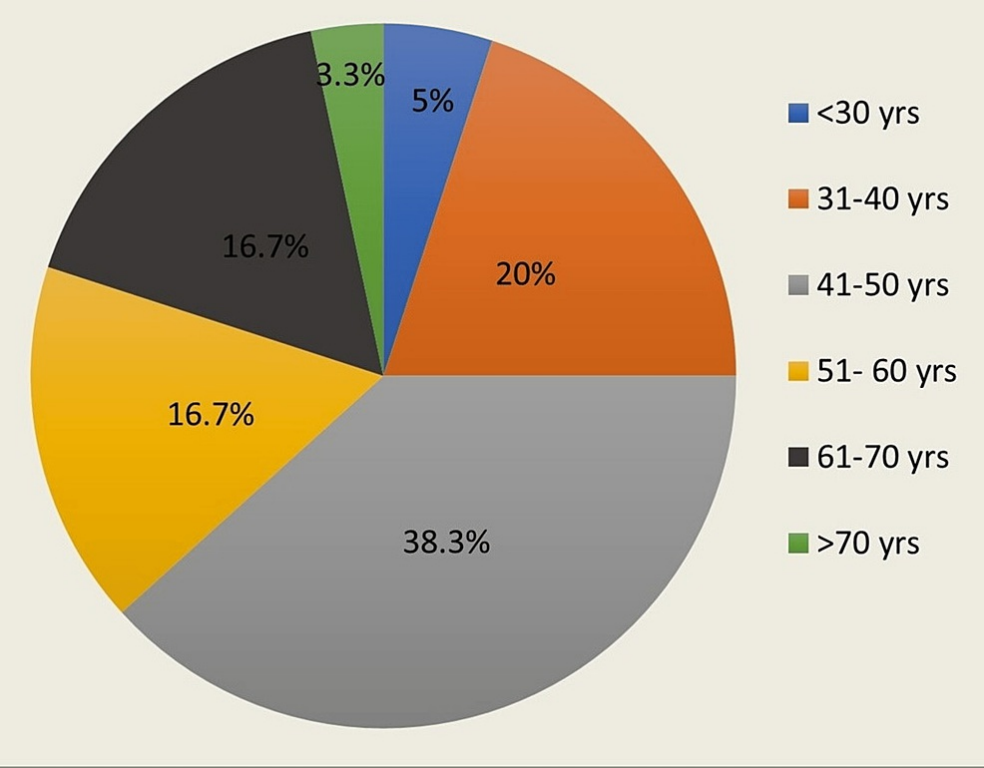
Pie chart showing the distribution of females in different age groups.

In the present study, 60 patients had undergone MRM. The patients were alternatively allocated into Group 1 (skin flap raised with electrocautery) and Group 2 (skin flap raised with a harmonic scalpel), with 30 patients in each group. The mean age of the patients in this study was 49.63+11.82 years (range 18-60 years). Figure 4 shows the distribution of patients in various age groups. It also shows that most patients belonged to the age group of 41-50 years.

Table 1 shows that the mean age group was higher for the patients with skin flaps raised by harmonic scalpel as compared to the patients with skin flaps raised by electrocautery.

**Table 1:** Inter-group comparison of mean age of cases studied. NS: Statistically non-significant.

Age (years)	Group 1 (Electrocautery) (n=30)		Group 2 (Harmonic scalpel) (n=30)		P-value
Age (years)	Mean	SD	Mean	SD	0.796^NS^
	48.63	11.89	49.43	11.94	

**Figure 5:**
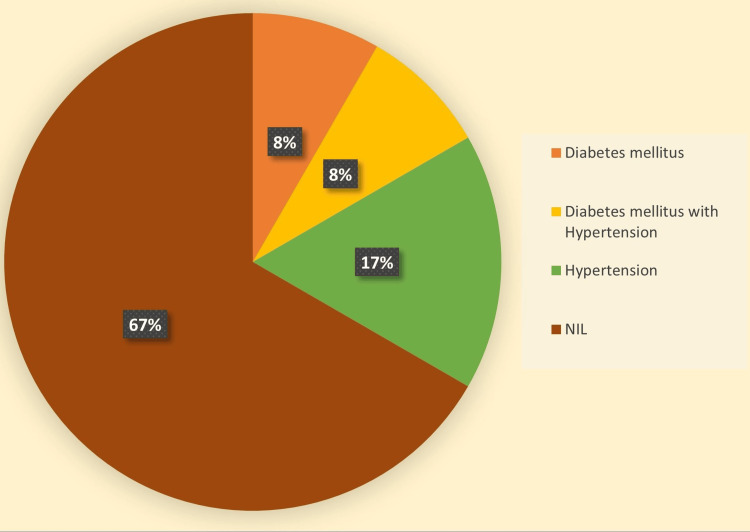
Pie chart showing the distribution of patients with different comorbidities. Hypertension was found to be the most common comorbidity among study subjects.

Figure 5 shows that out of the included study subjects with comorbidity, the most commonly associated comorbidity was increased blood pressure.

Table 2 and Table 3 show that the mean time taken for raising the skin flap and the total operative time was more in patients with the skin flap raised by a harmonic scalpel. Both these results were highly significant.

**Table 2:** Inter-group comparison of mean time taken for raising the skin flap among the cases studied. ***P-value <0.001 (P-value <0.05 is considered to be statistically significant).

Time for raising the skin flap (mins)	Group 1 (Electrocautery) (n=30)		Group 2 (Harmonic scalpel) (n=30)		P-value (Inter-group)
Time (mins)	Mean	SD	Mean	SD	Group 1 vs Group 2
	30.33	3.92	38.50	7.67	0.001^***^

**Table 3:** Inter-group comparison of mean operative time among the cases studied. **P-value <0.01 (P-value <0.05 is considered to be statistically significant).

Operative time (mins)	Group 1 (Electrocautery) (n=30)		Group 2 (Harmonic scalpel) (n=30)		P-value (Inter-group)
Time (mins)	Mean	SD	Mean	SD	Group 1 vs Group 2
	122.00	19.16	140.67	28.55	0.004^**^

Table 4 shows that the amount of intraoperative blood loss was significantly higher in patients with the skin flap raised by electrocautery. Table 5 indicates the amount of drain output in both groups which suggests to be more in the electrocautery group. This result was not significant. 

**Table 4:** Inter-group comparison of mean intra-operative blood loss among the cases studied. ***P-value <0.001 (P-value <0.05 is considered to be statistically significant).

Intra-operative blood loss (mL)	Group 1 (Electrocautery) (n=30)		Group 2 (Harmonic scalpel) (n=30)		P-value (Inter-group)
Blood loss (mL)	Mean	SD	Mean	SD	Group 1 vs Group 2
	178.33	21.06	138.50	28.53	0.001^***^

**Table 5:** Inter-group comparison of mean drain output on postoperative day 1 among the cases studied. NS: Statistically non-significant.

Drain output on postoperative day 1 (mL)	Group 1 (Electrocautery) (n=30)		Group 2 (Harmonic scalpel) (n=30)		P-value (Inter-group)
Drain output (mL)	Mean	SD	Mean	SD	Group 1 vs Group 2
	87.17	24.73	74.93	32.93	0.109^NS^

Table 6 shows the condition of the skin in the postoperative follow-up period. The number was found to be higher in the electrocautery group but this result was non-significant. Overall flap necrosis rate in our study was 10%. Table 7 shows the comparative analysis of patient factors with the incidence of skin flap necrosis. Intraoperative blood loss was found to be the most important factor for increased chances of flap necrosis among subjects. This also resulted in an increased duration of hospital stay for the management of skin flap necrosis. 

**Table 6:** Inter-group distribution of skin flap condition among the cases studied. NS: Statistically non-significant.

Condition of skin flap		Group 1 (Electrocautery) (n=30)		Group 2 (Harmonic scalpel) (n=30)		P-value (Inter-group)
		N	%	N	%	Group 1 vs Group 2
Postoperative day 3	Healthy	27	90.0	29	96.7	0.612^NS^
-	Unhealthy	3	10.0	1	3.3	
Postoperative day 7	Healthy	25	83.3	29	96.7	0.195^NS^
-	Unhealthy	5	16.7	1	3.3	
Postoperative day 30	Healthy	30	100.0	30	100.0	0.999^NS^
-	Unhealthy	0	0.0	0	0.0	

**Table 7:** Comparative analysis of patient factors to the development of skin flap necrosis. NA: Not applicable. **P-value <0.01 (P-value <0.05 is considered to be statistically significant).

Characteristics	Flap necrosis present (n=6)	Flap necrosis absent (n=54)	p-value
Harmonic scalpel used	1	29	NA
Electrocautery used	5	25	NA
Mean age (years)	49.00±13.62	49.04 ±11.75	0.99
Comorbidity	2	18	NA
BMI (kg/m2)	25.28 ±2.43	23.28 ±3.28	0.16
Neoadjuvant chemotherapy	2	17	NA
Time for raising flap (mins)	33.33 ± 6.06	34.53 ± 7.48	0.71
Total operative time (mins)	126.67 ± 26.01	131.85 ± 26.06	0.64
Intraoperative blood loss (ml)	193.33 ±15.06	154.53 ±31.02	0.004**
Hospital stay (days)	9.83 ±1.60	8.35 ±1.14	0.005**

Inter-group distribution of incidence of surgical site infection (SSI)

Of 30 cases studied in Group 1, one (3.3%) had SSI, and 29 (96.7%) did not have SSI on postoperative day 3. Of the 30 cases studied in Group 2, none had SSI on postoperative day 3. The distribution suggests that only one out of 30 patients in the electrocautery group developed surgical site infection which was statistically insignificant. Only one patient in the electrocautery group developed SSI while examined on postoperative day 3. The same patient was treated with continuation of antibiotics without any debridement and had no SSI while examined on postoperative day 7. No patient of either group had SSI when examined on postoperative day 30.

Inter-group distribution of incidence of seroma formation

Of 30 cases studied in the electrocautery group, 1 (3.3%) had seroma and 29 (96.7%) did not have seroma on postoperative day 3. Of the 30 cases studied in Group 2, none had seroma formation on postoperative day 3. Seroma formation was seen in a single patient in the electrocautery group but no seroma formation was seen in any patient of the harmonic scalpel group but the distribution was statistically insignificant on postoperative day 3. However, one patient in the harmonic scalpel group developed a seroma on examination on postoperative day 7. Both the patients with seroma formation were managed with repeated aspiration of the seroma collection and compression dressings. They were given antibiotics for a longer period along with anti-inflammatory drugs. Subsequently, both patients recovered with conservative management. Table 8 shows the total amount of drain output was non-significantly higher in patients with the skin flap raised with electrocautery. In contrast, the postoperative day of drain removal was higher in patients with the skin flap raised by the harmonic scalpel group as depicted in Table 9. This could be due to the fact that the drain was removed only when the total drain output was less than 30 ml over 24 hours.

**Table 8:** Inter-group comparison of mean total amount of drain among the cases studied. NS: Statistically non-significant.

Total amount of drain (mL)	Group 1 (Electrocautery) (n=30)		Group 2 (Harmonic scalpel) (n=30)		P-value (Inter-group)
Total amount (mL)	Mean	SD	Mean	SD	Group 1 vs Group 2
	310.83	88.93	298.20	127.87	0.659^NS^

**Table 9:** Inter-group comparison of the mean postoperative day of drain removal among the cases studied. NS: Statistically non-significant.

Postoperative day of drain removal (days)	Group 1 (Electrocautery) (n=30)		Group 2 (Harmonic scalpel) (n=30)		P-value (Inter-group)
Duration (days)	Mean	SD	Mean	SD	Group 1 vs Group 2
	6.83	0.75	7.43	2.27	0.174^NS^

## Discussion

Carcinoma breast is rarely encountered in children and adolescents, and after that, incidence increases with age to reach its peak in the 50-60 years age group. The mean age of patients included in our study was 49.63+11.82 yrs. The mean age in patients in whom electrocautery was used for skin flap raising was 48.63±11.89 years, and that of patients in whom harmonic scalpel was used for skin flap raising was 49.43±11.94 years. The age distribution did not differ significantly between the two study groups. In a study conducted by Mittal et al., the mean age of patients among the electrocautery and harmonic scalpel groups were 52±11.19 years and 50.36±11.04 years, respectively, which was very similar to our study [8]. The age-associated increased prevalence is due to prolonged exposure to estrogen hormones mainly, which can be in the form of early menarche, late menopause, late age of first childbirth, combined oral contraceptive pills, and hormone replacement therapy. Genetic factors and family history are also associated with early carcinoma occurrence in females [9]. Hypertension was the most common comorbid factor in our patient population, while some other patients also had diabetes mellitus or both.

In our study, the time taken to raise a skin flap using electrocautery was significantly lower than that of raising a skin flap using a harmonic scalpel. Burdette et al. concluded that the time taken for dissection using a harmonic scalpel and electrocautery was 33 mins and 31 minutes, respectively, which was statistically significant [4]. More time taken in the harmonic scalpel group is attributed to the fact that there was difficulty in getting the proper plane in the harmonic scalpel and a lack of experience in the use of the harmonic scalpel before the surgery. There is also ease of use of monopolar diathermy because of the smaller blade size. The mean operative time in patients with skin flaps raised using electrocautery was 122.00±19.16 mins, while the mean operative time in patients using harmonic scalpel was 140.67±28.55 mins. In a comparative study done by Mittal et al., the mean operative time was significantly greater in the group in which a harmonic scalpel was used compared to that in which electrocautery was used (140.40±29.96 vs. 99.80±24.00 min, P < 0.001) [8]. Our results were in contrast to the study conducted by Huang et al. and Deo et al., which showed no statistically significant difference [10,11]. In a retrospective cross-sectional study conducted by Memon et al., in the harmonic scalpel group, the mean operative time was 100.43±0.89 minutes, whereas, in the electrocautery group, it was 103.86±1.12 minutes with a significant difference (p=0.001) [12]. A harmonic scalpel takes slightly longer than electrocautery to divide the tissue as it cuts and coagulates simultaneously. This calls for patience and avoidance of undue traction on the surgical specimen, an almost instinctive reaction to the slower cutting rate [8,13].

The mean intraoperative blood loss in patients where the skin flap was raised using electrocautery was 178.33±21.06 mL. In contrast, the average intraoperative blood loss in patients where the skin flap was raised using a harmonic scalpel was 138.50±28.53 mL. This was in agreement with most of the studies like Mittal et al., Deo et al., and Kozomara et al., who reported that the harmonic scalpel causes less lateral thermal injury, seals and cuts the smaller blood vessels, which provides better hemostasis [8,11,14]. This results in lesser intraoperative blood loss. This benefit can help use a harmonic scalpel in patients with low preoperative hemoglobin to prevent undue intraoperative and postoperative blood transfusion. This will also prevent any blood transfusion-related reactions. The total drain output is lower in patients in which a harmonic scalpel raises the skin flap, but there is no statistical difference in drain output between the two groups. Similar results were seen in a study conducted by Burdette et al. with no statistically significant difference [4]. Studies by Khan et al., Mittal et al., Huang et al., and Deo et al. showed that harmonic scalpel decreases the drain volume, and the results are statistically significant [3,8,10,11]. The amount of inflammatory reaction in the operative field is reduced using a harmonic scalpel, which results in fewer lymphatic tissues being injured and less oozing surface produced in the operative field [15].

In our study, three out of 30 patients in the electrocautery group had flap necrosis on postoperative day 3, while one out of 30 patients in the harmonic scalpel group had flap necrosis. Five out of 30 patients had flap necrosis in the electrocautery group on examination on postoperative day 7, while one out of 30 patients in the harmonic scalpel group had flap necrosis. Lateral thermal injury is decreased using a harmonic scalpel compared to electrocautery, potentially reducing the flap necrosis rate. Patients with flap necrosis also have significantly more extended hospital stays than those without flap necrosis because the patients with flap necrosis require time for management in the form of regular dressing and monitoring the wound so that surgical debridement can be avoided. More is the duration of antibiotics in such patients.

In comparing seroma formation among both study groups, one out of 30 patients had seroma formation in the postoperative period. Studies conducted by Adwani et al., Mittal et al., Deo et al., and Galatius et al. did not show any statistical difference regarding the amount of seroma formation among the two groups similar to our study [5,8,11,16]. A study by Khan et al. and a metanalysis study by Huang et al. and Zhang et al. showed that a harmonic scalpel is superior to electrocautery in seroma formation after modified radical mastectomy [3,10,17]. Multiple aspirations can treat seroma following mastectomy of the collections. The frequency of aspirations is higher in patients where no drains are placed than in patients where drains are placed [18].

The mean time taken for drain removal postoperatively in the electrocautery group was 6.83±0.75 days, while that of the harmonic group was 7.43±2.27 days. In our study, the amount of drain output was less in the harmonic scalpel group, but the time taken for drain removal was still more extended in the harmonic scalpel group. This was because though the amount of drain was less in the harmonic scalpel group, it did not meet the criteria of drain removal, which was less than 30 ml over 24 hours, for which the drain had to be kept longer. This result was in contrast to the study conducted by Mittal et al., in which there was no statistical difference in the duration of drain removal among the two study groups [8]. None of the patients in the study groups developed any lymphedema, possibly because all patients were advised to undergo limb physiotherapy in the postoperative period. The mean duration of hospital stay in patients where the skin flap was raised by electrocautery was 8.57± 0.77 days, while the mean duration in the harmonic scalpel group was 8.43± 1.61 days. There was no statistical difference between the two groups regarding the mean duration of hospital stay. Studies also showed no statistically significant difference between the two groups concerning the duration of hospital stay [8,10,11].

The major limitation of our study is the small sample size due to the time-bound nature of the study. Another factor is the prevailing COVID-19 pandemic, which has led to fewer inpatients and surgeries conducted during that period. This may hinder the generalization of the results of the study. The operating surgeons' lesser experience in handling newer techniques like a harmonic scalpel compared to monopolar diathermy can be considered a confounding factor that resulted in increased duration of the procedure and intraoperative adverse events. The patients were only followed up for 30 days in this study, which might have resulted in missing out on delayed wound-related complications. This study has not considered the effect of neoadjuvant therapy on the intraoperative findings and postoperative outcomes. Follow-up trials with sufficiently large sample sizes and more extended follow-up periods are required to clear the cloud of confusion regarding the advantage of the use of a harmonic scalpel over monopolar diathermy for skin flap raising in mastectomy in breast cancer patients.

## Conclusions

The present study concludes that harmonic scalpel has limited advantages over electrocautery in mastectomy. Though a harmonic scalpel maintains hemostasis by decreasing the amount of intraoperative blood loss, it increases the duration of surgery. Harmonic scalpel also reduces the incidence of flap necrosis. However, this was found to be insignificant in this study. There was no significant difference between the two methods when factors like drainage volume, duration of drain removal, duration of hospital stay, and postoperative complications were considered. Still, the use of harmonic scalpels in breast surgeries is not cost-effective, and its use is not widespread in countries in which cost is a significant concern.
